# Superoxide imbalance triggered by Val16Ala‐SOD2 polymorphism increases the risk of depression and self‐reported psychological stress in free‐living elderly people

**DOI:** 10.1002/mgg3.1080

**Published:** 2019-12-31

**Authors:** Ivo Emilio da Cruz Jung, Ivana Beatrice Mânica da Cruz, Fernanda Barbisan, Alexis Trott, Lucien J. Houenou, Bárbara Osmarin Turra, Thiago Duarte, Raquel de Souza Praia, Ednea Aguiar Maia‐Ribeiro, Jaqueline da Costa Escobar Piccoli, Claudia Giugliano Bica, Marta Maria Medeiros Frescura Duarte

**Affiliations:** ^1^ Programa de Pós‐Graduação em Farmacologia Centro de Ciências da Saúde Universidade Federal de Santa Maria Santa Maria RS Brazil; ^2^ Programa de Pós‐Graduação em Gerontologia Universidade Federal de Santa Maria Santa Maria RS Brazil; ^3^ Departamento de Ciências da Vida Universidade Regional do Noroeste do Estado do Rio Grande do Sul Ijuí RS Brazil; ^4^ Biotechnology Department Forsyth Technical Community College Winston‐Salem NC USA; ^5^ Fundação Universidade Aberta da Terceira Idade (FUNATI) Manaus‐AM RS Brazil; ^6^ Programa de Pós‐Graduação em Ciências Farmacêuticas Universidade Federal do Pampa Uruguaiana RS Brazil; ^7^ Programa de Pós‐Graduação em Patologia Universidade Federal de Ciências da Saúde de Porto Alegre Porto Alegre RS Brazil

**Keywords:** depression, elderly, psychological stress, superoxide dismutase polymorphism

## Abstract

**Background:**

Oxidative stress and chronic inflammatory states triggered by a single‐nucleotide polymorphism (SNP) in superoxide dismutase manganese‐dependent gene (Val16Ala‐SOD2) have been associated with the risk of developing several chronic, nontransmissible diseases. However, it is still not clear whether the VV‐SOD2 genotype that causes higher basal superoxide anion levels has any impact on the risk for depression and self‐reported psychological stress in elderly people.

**Methods:**

In the present study, we tested this hypothesis using a case‐control study where depression was detected using the Geriatric Depression Scale‐15 (GDS‐15). A total of 612 Brazilian free‐living elderly subjects with a mean age of 67.1 ± 7.1 years old (number of controls, C = 497, and depressive individuals, D = 115) were included in this study. All participants had similar social, health, and lifestyle variables, with the exception of polypharmacy (≥5 medicines daily intake), which was higher in the D group, compared to C subjects.

**Results:**

Our results showed that the VV‐SOD2 genotype significantly increased the risk for depression and psychological stress in the elderly subjects, independently of sex/gender, age, and other prior diseases and health indicators (depression risk = 1.842, 1.109–3.061 95% CI, *p* = .018). VV‐subjects also had a higher daily intake of antidepressants, anxiolytics, and anti‐inflammatory drugs than A‐allele subjects.

**Conclusion:**

Our findings support the hypothesis that genetically induced oxidative superoxide‐hydrogen peroxide imbalance may be involved in an increased risk for developing depression and psychological stress in free‐living elderly people without other chronic nontransmissible diseases.

## Graphical abstract

Our results showed that the VV‐SOD2 genotype significantly increased the risk for depression and psychological stress in the elderly subjects. VV‐subjects also had a higher daily intake of antidepressants, anxiolytics, and anti‐inflammatory drugs. The findings support the hypothesis that genetically induced oxidative superoxide‐hydrogen peroxide imbalance may be involved in an increased risk for developing depression and psychological stress in free‐living elderly people.

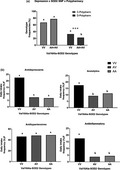

## 1 INTRODUCTION

Depression and psychological stress are highly prevalent in contemporary societies, mainly in elderly people (Demyttenaere et al., 2009; Kok & Reynolds, 2017; Niraula, Sheridan, & Godbout, 2017). Moreover, these conditions can affect the well‐being and increase the risk of certain chronic nontransmissible diseases, such as cancer and cardiovascular morbidities (Bortolato et al., 2017; Zhang, Chen, & Ma, 2018). The convergence of psychological stress, depression, and other chronic nontransmissible diseases may be related to the establishment of inflammatory oxidative states present in all these conditions (Halaris, 2017; Kruse & Strouse, 2015).

Numerous studies have consistently suggested the existence of a bidirectional relationship between oxidative stress inflammation and these conditions (Demyttenaere et al., 2009; Liu et al., 2018; Prenderville, Kennedy, Dinan, & Cryan, 2015; Straub & Cutolo, 2018). Previous investigations have suggested that oxi‐inflammatory states triggered by depression and/or chronic psychological stress appear to be associated with the elevation of reactive oxygen species (ROS) (Lopresti, Maker, Hood, & Drummond, 2014), including superoxide anion levels produced by NADPH oxidase activation or some impairment in superoxide dismutase (SOD) enzymes (Seo et al., 2012; Uchihara, Tanaka, Asano, Tamura, & Mizushima, 2016; Xie et al., 2017).

Genetic studies involving the imbalance of superoxide‐hydrogen peroxide associated with a single‐nucleotide polymorphism (SNP) located in the *SOD2* (OMIM: 147460), manganese‐dependent enzyme gene (Val16Ala‐SOD2, rs4880) have suggested an association of both AA‐ and AV‐genotypes of this SNP with some chronic nontransmissible diseases. This association correlates with a superoxide‐hydrogen peroxide imbalance that involves a higher efficiency of *SOD2* in the AA‐genotype; this generates elevated basal hydrogen peroxide levels, and a lower efficiency of *SOD2* in the VV‐genotype generating elevated basal superoxide levels in carriers (Bresciani, Cruz, & González‐Gallego, 2015; Bresciani, González‐Gallego, da Cruz, de Paz, & Cuevas, 2013). Specifically, the VV‐genotype has been linked to chronic inflammatory states with higher levels of proinflammatory cytokines, such as the interleukins, IL‐1 and IL‐6, tumor necrosis factor‐alpha (TNF‐α), and anti‐inflammatory interleukin IL‐10 (Barbisan, Azzolin, & Ribeiro, 2017; Duarte et al., 2016; Montano et al., 2012). Flores et al. (2017) also suggested that the VV‐genotype could increase the risk for hypercholesterolemia and higher glucose levels in patients in the late phase of stroke (>6 months).

However, studies correlating the Val16Ala‐SOD2 SNP with depression are still inconclusive. While some studies have not found an association between this SNP and depression (Elbozan Cumurcu et al., 2013; Pae et al., 2006), other investigations have described a higher frequency of both VV‐genotypes and depression in female subjects (Gałecki et al., 2010) when examining the severity of depression in patients with chronic obstructive pulmonary disease (Pietras et al., 2010) as well as in adult patients with depression (Wigner et al., 2018).

Considering that oxidative stress and chronic inflammation are two processes that increase during the aging process (Picca et al., 2018), the present study postulated that the risk of both depression and psychological stress in elderly people may be influenced by superoxide‐hydrogen peroxide imbalance triggered by the Val16Ala‐SOD2 SNP.

## 2 MATERIALS AND METHODS

### 2.1 General study design

To investigate the possible effects of genetically determined superoxide‐hydrogen peroxide imbalance on depression and self‐reported chronic psychological stress, we performed a case‐control analysis that examined the association between the Val16Ala‐SOD2 SNP and depression, and self‐reported psychological stress in the free‐living elderly and subjects without cognitive impairment. All participants provided written informed consent, and this protocol was approved by the Human Ethics Committee of the Federal University of Santa Maria (number 23081.009087/2008). The work described in the present paper has been carried out in accordance with The Code of Ethics of the World Medical Association (Declaration of Helsinki).

### 2.2 Genetic Analyses

The Val16Ala‐SOD2 SNP of all study participants was analyzed from DNA samples using standard polymerase chain reaction and restriction fragment length polymorphism (PCR‐RFLP) techniques, previously described by Montano et al. (2009). Briefly, after obtaining DNA from total peripheral blood leukocyte extraction, *SOD2* genotyping was performed following amplification of a 110‐bp fragment of the human *SOD2*: 5′‐ACCAGCAGGCAGCTGGCGCCGG‐3′ (sense‐strand) and 5′GCGTTGATGTGAGGTTCCAG‐3′ (antisense‐strand). Further, PCR products (10 µl) obtained after amplification were digested with HaeIII (15 U; at 37°C, for 6 hr, Gibco Inc.) and two digested products (23 and 85 bp) were visualized on a 6% agarose gel (Amersham Biosciences Inc.) stained with ethidium bromide.

### 2.3 Case‐control protocol

The current protocol included a Brazilian elderly free‐living community with an average age of 67.1 ± 7.1 years, (minimum = 60 years and maximum = 82 years) enrolled in third age‐social groups or in geriatric social support services (Gravataí city, State of Rio Grande do Sul, Brazil). At the time the study was conducted, there were 27,453 elderly people in the city of Gravataí. Sample size calculation considering 95% confidence and a 5% margin of error estimated the inclusion of 379 elderly. However, considering a prevalence of depression estimated to be 5.8% for population, it was decided to include all elderly participants in the study who did not meet any exclusion criteria. We considered that this population does not present any significant ethnic isolation been appropriate for epidemiological studies on aging based in previous investigations performed by Da Cruz et al. (2003) and Parra et al. (2003). We did not include subjects who were bedridden, hospitalized, or who did not participate regularly in social activities outside their homes. Subjects diagnosed with cognitive decline or immobility problems due to chronic morbidities—such as stroke, Parkinson's disease, hypercholesterolemia, morbid obesity, and some types of cancer—were excluded from the study, since these morbidities are confounding factors associated with high rates of depression. Therefore, from the original databank of 1,058 elderly people previously studied by Montano et al. (2009), 612 subjects were included in the present analysis. Notably, as Brazil is a middle‐income country, the World Health Organization considers elderly people to be those ≥60 years old (WHO, 2015).

Depression in the elderly sample was diagnosed using the Geriatric Depression Scale‐15 (GDS‐15), previously validated in Portuguese (Almeida & Almeida, 1999), which is able to achieve early detection of depression in primary care and other health care settings. GDS is an instrument broadly used to diagnose depression in elderly people, and several studies have demonstrated that GDS offers valid and reliable measures for the evaluation of depressive disorders (Dorow et al., 2018). The Portuguese version of the GDS with 15 questions (GDS‐15) offered valid measurements for the diagnosis of major depressive episodes according to the International Classification of Diseases (ICD‐10) and the Diagnostic and Statistical Manual of Mental Disorders, 4th edition (DSM‐IV) criteria using a 5/6 cutoff point (noncase/case) (Almeida & Almeida, 1999), which was also predicted previously by Hermann et al. (1996). The 5/6 cutoff point (noncase/case) for the GDS‐15 produced sensitivity indices of 85.4% and a specificity of 73.9% for the diagnosis of depressive episodes, according to the ICD‐10 (Almeida & Almeida, 1999). An evaluation of 23 studies performed by Pocklington, Gilbody, Manea, and McMillan (2016) indicated that a cutoff score of 5 points was appropriate for the GDS‐15. However, it is important to be cautious about the classification of elderly depressive episodes using this cutoff point. Some studies, such as the one performed by Sugishita, Sugishita, and Hemmi (2017), suggested that a higher cutoff point (6/7) could be more accurate for diagnosis of a depressive episode in elderly people. Therefore, in the present study, we decided to use the 6/7 cutoff point.

Self‐perception of current psychological stress was also considered in our analysis. Two physicians and one psychologist determined the GDS score and self‐perception of psychological stress, whereas health professionals conducted a structured interview with questions regarding social, health, and lifestyle variables. Subjects with chronic or noncontrolled diseases that could influence the results were excluded. Since the study includes genetic variables, the subjects were recruited based on a random selection of Brazilians of European ancestry from State of Rio Grande do Sul. All subjects provided written informed consent, and our protocol was approved by the Human Ethics Committee of the Federal University of Santa Maria, Santa Maria, RS, Brazil (number 23081.009087/2008).

Demographic and social variables were considered covariates in the study. Moreover, the following co‐variables that could exert some influence on the association of Val16Ala‐SOD2 SNP with depression were considered in the analysis, since previous reports have shown an association between these variables and the polymorphism studied here. These variables include, obesity determined by body mass index (BMI, Kg/m^2^) (Hernández‐Guerrero et al., 2016; Montano et al., 2009) or obesity‐related metabolic markers (Becer & Çirakoğlu, 2015), hypercholesterolemia (Chen et al., 2012; Duarte et al., 2010; Duarte et al., 2016; Flores et al., 2017), and diabetes mellitus type 2 and its complications (Gottlieb et al., 2005; Huang, Lyu, Liu, Chen, & Wang, 2017; Möllsten, Jorsal, Lajer, Vionnet, & Tarnow, 2009; Li et al., 2015; Pourvali, Abbasi, & Mottaghi, 2016; Rizvi, Raza, & Mahdi, 2014).

Furthermore, the following three co‐variables were also considered despite a lack of specific associative studies between them and Val16Ala‐SOD2 polymorphism: hypertension, smoking, and polypharmacy. Previous reports describe an association between the Val16Ala‐SOD2 SNP and cardiovascular diseases (Flores et al., 2017; Fujimoto, Kobayashi, Ogasawara, Yamakado, & Ohno, 2010; Fujimoto et al., 2008; Kakko et al., 2003; Karahalil, Elkama, & Orhan, 2017; Souiden et al., 2016), for which hypertension and smoking habits are important risk factors. Regarding polypharmacy, consistent evidences exist supporting an association between certain pharmacological drugs and body weight modulation, including antidepressants (Abosi, Lopes, Schmitz, & Fiedorowicz, 2018) and antipsychotic drugs, which are sometimes prescribed to depressive elderly patients (Dayabandara et al., 2017).

### 2.4 Statistical analyses

Data analysis was performed with SPSS (version 22.0.1; SPSS Inc.). Initially, the Hardy–Weinberg equilibrium was tested using a Chi‐squared test in which observed and expected genotype frequencies were compared. Univariate association of *SOD2* genotypes and V‐ or A‐allele frequencies with depression and self‐reported psychological stress was also determined using Chi‐squared or Fisher test. Quantitative variables were compared among genotypes using analysis of variance (ANOVA) followed by a Bonferroni post hoc test. Considering that the elderly can present some differences related to sex/gender, age, or previous disease, and because some chronic diseases have previously been associated with the Val16Ala‐SOD2 SNP, such as obesity and dyslipidemia, a multivariate analysis was performed using a logistic regression (backward stepwise Wald method) to determine the effect of these variables on the association between depression, psychological stress, and the *SOD2* SNP. Odds ratio values and 95% confidence intervals were also calculated. The alpha value was set at 0.05, and all *p*‐values were two‐tailed.

## 3 RESULTS

Out of the 612 elderly subjects included in the present analysis, 115 (18.8%) were diagnosed with depression (D group), whereas 497 (81.2%) were not and used as controls (C group). The mean age of the two groups was virtually similar (D = 66.9 ± 6.5 and C = 67.2 ± 7.0 years, *p* = .728). Social, health, and lifestyle variables were also similar between the two groups. However, polypharmacy, that is, daily intake of ≥5 prescribed drugs, was higher in the D subjects, compared to the C group. Furthermore, subjects in group D also presented a higher prevalence of self‐reported psychological stress than the subjects in group C.

Comparison of the genotypic frequency of the Val16Ala‐SOD2 SNP in groups C and D elderly subjects is presented in Table 1, which shows that the subjects in group D had a significantly higher VV‐frequency than the healthy individuals in group C. Further analysis revealed a significant association between the VV‐genotype and depression, compared to A‐allele carriers. Therefore, this association presented a potential recessive pattern in the population studied here. Elderly subjects who self‐reported psychological stress also had a significantly (*p* = .02) higher frequency of VV‐genotypes (31.3%, *n* = 47) than controls (19.8%, *n* = 51). The relative risk (RR) for the D group of presenting VV‐genotypes was 1.564 (range: 1.146 to 2.134) times higher than the RR of C controls. A multivariate analysis showed that the association between depression and the VV‐genotype was independent of sex, age, and several other health variables, as shown in Table 1.

**Table 1:** Genotype and allele frequencies of Val16Ala‐SOD2 SNP in healthy control (C) and depressive (D) elderly subjects

Genetic	C subjects % (*n*)	D subjects % (*n*)	*p*
Genotypes			
VV	21.1 (105)	33.0 (38)	.014
AV	62.8 (312)	49.6 (57)	
AA	16.1 (80)	17.4 (20)	
Allele frequency			
V	0.525	0.665	
A	0.475	0.335	
V‐allele dose effect			
AA + AV	78.8 (392)	67.0 (77)	.006
VV	21.2 (105)	33.0 (38)	

Note

Statistical comparisons were performed by Chi‐squared test.

**Figure 1:**
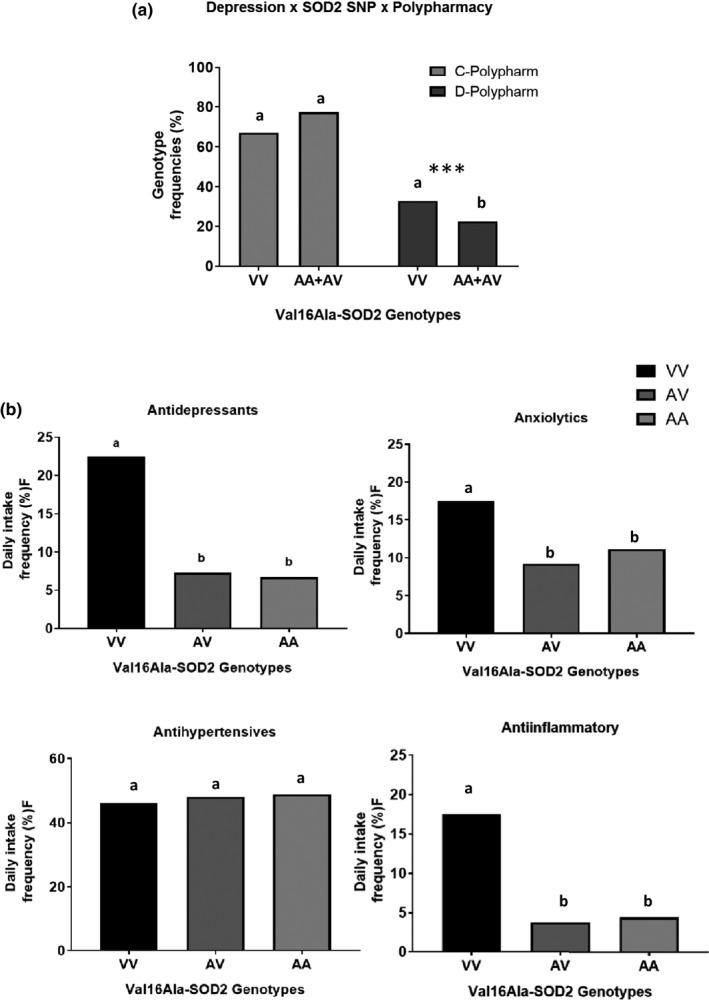
Val16Ala‐SOD2 genotype distribution in control (C) and depressive (D) elderly subjects who presented (a) polypharmacy (daily intake of ≥5 medicines) and (b) hypertension (HAS) disease. The comparison was performed by univariate Chi‐squared analysis. ***p < .01. In logistic regression multivariate analysis, polypharmacy and hypertension were associated with depression diagnosis, but did not significantly influence the association between the VV‐genotype and depression

Additional analysis showed that VV‐depressive subjects had a higher frequency of polypharmacy than AA‐ and AV‐depressive subjects (Figure 1a). A complementary analysis was conducted to analyze antidepressants and other drugs used daily by 198 elderly subjects with different Val16Ala‐SOD2 genotypes (Figure 1b). This analysis was performed in 198 subjects who were able to report what drugs they ingested daily. The VV‐carriers reported a significantly higher frequency of daily intake of antidepressants (*p* = .032) and anti‐inflammatory drugs (*p* = .011) than A‐allele carriers. The RR of VV‐subjects using antidepressants compared to A‐allele subjects was 3.628 (95% CI = 1.222–10.773) independent of sex, age, BMI, and use of hypolipemic agent. The RR of VV‐subjects using anti‐inflammatory drugs compared to A‐allele subjects was 5.636 (95% CI = 1.638–9.385) independent of sex, age, BMI, and hypolipemic agent use. However, the frequencies of antihypertensive and anxiolytic drugs, including sleeping remedies, were similar among subjects carrying different *SOD2* genotypes (see Figure 1b).

In this analysis, a total of five VV‐carriers (12.8%) reported the use of statins, whereas in AV‐subjects, the use of this drug was reported by six subjects (5.8%) and by one AA‐subject (2.3%). These results were not significantly different among *SOD2* genotype groups (*p* = .155). The number of elderly subjects reporting the use of hypoglycemic drugs was also similar (*p* = .748) and restricted (*n* = 6, 3.1%) in the subsample analyzed here.

## 4 DISCUSSION

The present investigation found a significant association between depression and self‐reported current psychological stress and the VV‐genotype of the Val16Ala‐SOD2 SNP. These results were independent of sex, age, and several other health variables such as diabetes, dyslipidemia, and obesity (Table 2). However, hypertension and polypharmacy were significantly associated with depression (Table 3). Despite methodological constraints related to case‐control studies, our results suggested a potential association of genetic SNP associated with a lower efficiency in SOD2 enzyme with neuropsychiatric conditions prevalent in elderly subjects. Consequently, some detailed considerations about these results are discussed below.

**Table 2:** Characteristics of elderly subjects with (D) and without (C) depression

Variables	C subjects % (*n*)	D subjects % (*n*)	*p*
Age groups (years)			
60–64	37.6 (187)	37.4 (43)	.907
65–69	27.4 (136)	30.4 (35)	
70–74	20.9 (104)	20.9 (24)	
75–79	8.9 (44)	7.8 (09)	
>80	5.2 (26)	3.5 (04)	
Sex			
Male	21.7 (108)	19.1 (22)	.539
Females	78.3 (389)	80.9 (93)	
Marital status			
Married/partnership	37.9 (189)	33.0 (38)	.102
Single	6.1 (30)	3.5 (04)	
Widow	46.1 (229)	58.3 (67)	
Divorced	9.9 (49)	06 (5.2)	
Education (years)			
0 < 3	11.3 (56)	13.0 (15)	.879
3 < 8	35.3 (175)	33.9 (39)	
>8	53.4 (266)	53.0 (61)	
Diabetes mellitus 2	11.5 (57)	15.7 (18)	.348
Hypertension	56.5 (281)	66.1 (76)	.061
Dyslipidemia	47.3 (235)	49.6 (57)	.659
Obesity	38.6 (192)	38.3 (44)	.941
Metabolic syndrome	12.9 (64)	13.9 (16)	.767
Polypharmacy	23.4 (116)	35.7 (41)	.017*
Smoking habit (current/former)	26.3 (129)	35.2 (103)	.112
Self‐reported psychological stress	11.9 (59)	98.0 (116)	<.0001*

Note

C = control group; D = group with depression diagnosis; Subject numbers are shown as (*n*).

Statistical univariate comparison between two elderly groups was performed by Chi‐squared or Fisher's exact test.

* Statistically significant difference between the C and D groups.

**Table 3:** Multivariate logistic regression analysis to determine the influence of some healthy conditions in the association between VV‐SOD2 genotype and depression in elderly free‐living community

Variables	Wald	Risk	95% CI	*p*
VV‐genotype	5.562	1.842	1.109–3.061	0.018*
Sex	0.150	1.119	0.635–1.971	0.698
Age	0.756	0.385	1.015–1.049	0.982
Smoking habit	2.660	1.518	0.919–2.506	0.103
Polypharmacy	6.932	0.517	0.316–0.845	0.008*
Obesity	0.041	0.952	0.594–1.527	0.839
Dyslipidemia	1.914	1.383	0.874–2.189	0.167
Diabetes mellitus 2	0.814	1.359	0.698–2.645	0.367
Hypertension	8.756	2.019	1.268–3.215	0.003*

Note

Multivariate analysis: logistic regression Backward Wald method.

Abbreviation: CI, confident interval.

* Statistically significant difference.

Depression is a psychiatric disease that affects a large number of people, especially the elderly, and is highly associated with suicide (Bachmann, 2018). Data from the World Mental Health Survey, conducted in 17 countries, estimated that one in 20 people present at least one episode of depression throughout their lifetime (Wang et al., 2007). However, known cases of depression do not provide sufficient explanation for the underlying pathophysiology. Evidence suggests that chronic psychological stress is an important risk factor associated with the development of depression, since it is implicated in the induction of multiple behavioral, neurochemical, and biological alterations (Tagliari et al., 2010). Therefore, the association between VV‐SOD2 and depression described in previous studies (Gałecki et al., 2010; Pietras et al., 2010) and in the present investigation could be epidemiologically relevant.

The plausible association between *SOD2* genetic imbalance and depression is based on well‐documented evidence that chronic psychological stress is associated with ROS overproduction and the pathogenesis of depression (Floyd, Towner, He, Hensley, & Maples, 2011; Michel et al., 2007; Siegrist & Sies, 2017; Wei et al., 2009). Moreover, there are some investigations, such as one performed by Maes, Galecki, and Chang (2011), that reported an association between depression and impairment of antioxidant status. Therefore, the results described here regarding case‐control analysis strongly support the hypothesis that basal oxidative stress conditions associated with the *SOD2* SNP may have some major influence on the risk of depression in the elderly. From our results, it is possible to infer that basal oxidative imbalance associated with a genetic polymorphism could increase the susceptibility of some people in developing depressive states during old age. However, in the present investigation, we did not performed complementary investigations on levels of some oxidative blood markers that could corroborate the association between VV‐genotype and depression. In fact, it is not always possible to establish a direct association between serum levels of oxidative markers including lipoperoxidation, protein carbonylation, and DNA damage since the superoxide‐hydrogen peroxide imbalance associated with the SOD2 enzyme occurs within mitochondria. However, in some previous studies conducted by our research group, we have even found some level of association between Val16Ala‐SOD2 genotypes and oxidative markers. This was the case for analyzes related to the association between hypercholesterolemia and polymorphism or the pharmacogenetic effect of this polymorphism on rosuvastatin response (Duarte et al., 2010; Duarte et al., 2016). In both cases, the research volunteers were chosen because they had a very similar lifestyle, health, and dietary profile. However, in studies involving the elderly, this type of selection is very difficult to conduct as a result of the heterogeneity of this population group. For this reason, we chose not to include serum analyzes related to the oxidative profile.

Moreover, despite the potential association between the VV‐genotype, which has higher basal levels of superoxide anion, it is important to point out that investigations involving this SNP in psychiatric and psychological conditions are still unclear and controversial. From the literature, we found three previous investigations that suggested some association between depression and the VV‐genotype (Gałecki et al., 2010; Pietras et al., 2010; Wigner et al., 2018). Other studies involving the potential association between the Val16Ala‐SOD2 SNP and mood disorders failed to show a significant association between this SNP and major depressive and/or bipolar disorders. However, these studies were performed with small sample sizes (Elbozan Cumurcu et al., 2013; Pae et al., 2006).

Moreover, it is not easy to establish association studies involving the genetics of oxidative metabolism, since there are several potential intervening factors that can act as attenuators or enhancers of oxidative stress associated with psychiatric diseases. For this reason, we tried to perform a prescreening of our sample population, excluding elderly subjects who presented cognitive impairment, physical and psychological frailty, and previous chronic or noncontrolled diseases. Under these conditions, we opted to investigate depression in an elderly free‐living community that participated in organized social groups. This selection could be relevant in the observation of a significant association between the *SOD2* SNP and depression described in the present report.

There are several biological and psychological theories explaining the causes of depression including the hypothesis of an active inflammatory process associated with oxidative metabolism imbalance (Jeon & Kim, 2018). In relation to inflammatory response, there is consistent number of studies indicating that superoxide anion is important in this process. Superoxide anion mainly produced by NAD(P)H oxidases is present in all cell types participating in inflammation (leukocytes, endothelial, and other vascular cells) (Zeng, Miralda, Armstrong, Uriarte, & Bagaitkar, 2019). However, basal uncontrolled superoxide concentration may lead to toxic effects, when produced at high levels during oxidative burst. This process has been associated with VV‐genotype that is associated with higher risk of chronic inflammatory conditions including hypercholesterolemia, obesity, and cardiovascular diseases, such as stroke (Barbisan et al., 2017; Bresciani et al., 2015; Pascotini et al., 2018) and also depression and other mood disorders (Maes et al., 2019; Valvassori et al., 2018). As pointed out by Kalinichenko's review (2019), investigations described that psychological stress and mood disorders, especially depression, can result in abnormal immune responses accompanied by abnormal levels of ROS in the red blood cells, mononuclear cells, cerebrospinal fluid, and the brain. In this process, superoxide is considered a key molecule of inflammatory activation, and previous investigations suggested that its imbalance could directly affect the oxi‐inflammatory metabolic patterns. In order to test this hypothesis, Barbisan et al. (2017) evaluated in vitro inflammatory response of peripheral blood mononuclear cells carrying different Val16Ala‐SOD2 genotypes. Results showed that VV‐cells were associated with higher proinflammatory cytokine levels indicating a genetic role in chronic oxi‐inflammatory processes. Therefore, basal higher superoxide levels in VV‐subjects could explain the association between this genotype and depression previously described by Gałecki et al. (2010), and in the present study.

Despite difficulties in evaluating the impact of basal oxidative imbalance on depression risk in elderly people, the findings summarized here are consistent with a recent investigation published by Wigner et al. (2018) describing a significant association between the Val16Ala‐SOD2 SNP and depression in adult subjects. Similar to what was observed in the elderly subjects in our study, Wigner et al. (2018) showed that subjects with the VV‐genotype presented a higher risk of depression diagnosis than AA‐ and AV‐subjects. The authors concluded that their results supported the hypothesis that oxidative and nitrosative stress are involved in the pathogenesis of depressive disorders. Basal alteration in oxidative metabolism triggered by the VV‐genotype leads to the increase of superoxide anion levels, a key molecule in inflammatory processes.

The potential association between higher superoxide anion levels and psychological stress and depression is also indirectly supported by a study in humans performed by Zuccarella‐Hackl et al. (2016). These authors described an association between higher superoxide anion levels and pathogenesis of coronary artery disease, especially in patients with Type D personalities, that is, those with a tendency to experience negative emotions and to inhibit their expression in a social context.

Free‐living elderly people as a group probably experience several instances of “loss”, including the death of family members or friends, the loss of social status associated with retirement, and even functional losses. Thus, elderly individuals who do not have some type of depression or more prominent depressive symptoms may be considered more resilient, whereas depressed individuals may be considered more variable. It is important to point out that we concentrated our analysis on this elderly group since elderly people who present devastating chronic, nontransmissible diseases or cognitive dysfunction have a high predisposition for developing depressive disorders as a secondary morbidity. Among these diseases are some neurodegenerative morbidities such as Parkinson's, coronary diseases, including myocardial infarction, and even some types of cancer (Nasca, Davis, Bigio, Sandi, & McEwen, 2017).

Finally, it is important to comment on some aspects of the methodological approaches used in this study, specifically the use of GDS as a tool to evaluate depression. Accurate diagnosis is essential for the management of elderly depression in primary care. For this reason, several scales have been developed, including the GDS‐15, the Hospital Anxiety and Depression Scale, and the Structured Clinical Interview for DSM‐V criteria. However, a recent study showed that there were high levels of inconsistency among depression diagnosis performed by different tools when more than 1,000 75+ years old patients were concomitantly assessed with these measurement scales. Despite these differences, GDS was the tool that achieved results close to those obtained by a general practitioner (GP) based on the DSM‐V. Whereas the GP estimated there to be 24.3% depressive elderly subjects, the GDS‐15 estimated there to be 21.8% (Dorow et al., 2018). Moreover, the GDS was previously validated in Brazil and is broadly used in geriatric anamneses for the screening and diagnosis of depression (Almeida & Almeida, 1999).

However, there are some considerations that need to be commented on regarding the GDS‐15 tool. Many instruments are available to measure depression, including the GDS with 30 questions, first created by Yesavage et al. (1982), which has been tested and used extensively with elderly subjects. A shortened GDS form developed in 1986 contains 15 questions of which 10 questions indicate the presence of depression when answered positively, while five questions indicate depression when answered negatively. When the GDS‐15 was created, older subjects with scores of 0–4 were considered nondepressives, those with a score of 5–8 were considered to have mild depression, 9–11 indicated moderate depression, and 12–15 indicated severe depression. However, authors such as Greenberg (2012) commented that these cutoff points could vary depending on age, education, and complaints. For this reason, studies generally use the GDS‐15 as a screening tool to identify depressive older people, and not to identify the intensity of their psychiatric condition.

Moreover, there are some concerns about the GDS‐15 cutoff points with respect to depression diagnosis. Most studies consider 4/5 points to be an appropriate cutoff (Pocklington et al., 2016), whereas other studies suggest that 6/7 could be a better cutoff (Sugishita et al., 2017). Because of this, we opted to exclude from our main analysis subjects who could present an overlapping diagnosis in relation to noncase and case distributions. Not excluding these subjects could compromise the results and their interpretations, since we would be including individuals with intermediate punctuation. We understand that this strategy is a significant methodological constraint; nonetheless, we have opted to be more cautious regarding this issue in order to accurately test our main hypothesis pertaining to the association between depression and genetic oxidative imbalance.

Another factor that may be considered limiting in the present study is the following question: we found 98% of self‐declaration of psychological stress in depressive subjects, there is really an association of VV‐SOD2 genotype and stress, or there is a bias of the sample selection, since almost all depressive subjects are also stressed subjects? This question is really important to consider. However, we believe the association between the VV‐genotype psychosocial stress and/or depression is plausible and true, and not just a sample selection bias based in the following considerations: Evidences indicate that the hyperactivity of the HPA axis that induces changes in the immune system is responsible for some of the behavioral and biochemical changes that are also typical in depression (review in Kalinichenko, Kornhuber, & Müller, 2019). Therefore, epidemiological and clinical studies have suggested strong association between psychosocial stress and depression. However, previous report estimated that only approximately 3%–5% of human individuals develop depression/anxiety after stressful life events (Wada et al., 2013). Based on these estimative, we find it important to question older people on their self‐perception of chronic stress. In both cases, elderly self‐reported psychosocial stresses and depressed elderly, we found higher frequency of VV‐genotype indicating that this genetic factor could affect the susceptibility for mental conditions.

## 5 CONCLUSIONS

The results described in the present report, strongly suggest the association between Val16Ala‐*SOD2* SNP and risk of depression and self‐reported psychological stress in elderly subjects, independent of other prevalent noncommunicable chronic diseases. Considering previous evidence, this association could be related to the increase of basal oxidative stress and/or inflammatory states prevalent in VV‐subjects. This association also seems to influence the daily intake of prescribed drugs by older subjects.

## CONFLICT OF INTEREST

The authors declare no conflict of interest.

## AUTHORS CONTRIBUTION

Ivo Emilio da Cruz Jung—Mainly responsible for execution of study, writing the manuscript. Ivana da Cruz—Co‐responsible for execution of study, data collection, writing the manuscript study design and statistical analysis. Alexis Trott—Standardization of Val16Ala‐SOD2 genotyping, writing the manuscript, corresponding author. Fernanda Barbisan—Data collection. Lucien Houenou—Review of methodological design, statistical analysis, English editing manuscript. Bárbara Osmarin Turra—Genotyping, databank organization. Thiago Duarte—Genotyping, databank organization. Raquel de Souza Praia—Data collection. Ednea Aguiar Maia‐Ribeiro—Data collection. Jaqueline da Costa Escobar Piccoli—Data collection. Claudia Giugliano Bica—Population data collection. Marta Maria Medeiros Frescura Duarte—Scientific coordinator of Project, responsible for study design and statistical analysis.
